# Predictive Validity of Mortality after Surgically Treated Proximal Femur Fractures Based on Four Nutrition Scores—A Retrospective Data Analysis

**DOI:** 10.3390/nu15153357

**Published:** 2023-07-28

**Authors:** Domenik Popp, Arastoo Nia, Gregor Biedermann, Lukas Schmoelz, Sara Silvaieh, Thomas M. Tiefenboeck, Stefan Hajdu, Harald K. Widhalm

**Affiliations:** 1 Clinical Division of Traumatology, Department of Orthopedics and Trauma Surgery, Medical University of Vienna, 1090 Vienna, Austria; domenik.popp@meduniwien.ac.at (D.P.); arastoo.nia@meduniwien.ac.at (A.N.); gregor.biedermann13@gmail.com (G.B.); lukas.schmoelz@meduniwien.ac.at (L.S.); thomas.tiefenboeck@meduniwien.ac.at (T.M.T.); stefan.hajdu@meduniwien.ac.at (S.H.); 2 Department of Neurology, Medical University of Vienna, 1090 Vienna, Austria; sara.silvaieh@meduniwien.ac.at

**Keywords:** hip fracture, risk prediction, mortality, MNA, GMS, MUST, NRA, malnutrition

## Abstract

Background: Hip fractures are becoming a growing concern due to an aging population. The high costs to the healthcare system and far-reaching consequences for those affected, including a loss of independence and increased mortality rates, make this issue important. Poor nutritional status is a common problem among geriatric patients and is associated with a worse prognosis. Nutritional screening tools can help identify high-risk patients and enable individualized care to improve survival rates. Material and methods: This retrospective study investigates four nutritional scores and laboratory parameters’ predictive significance concerning postoperative mortality after surgical treatment of proximal femur fractures at 1, 3, 6, and 12 month/s for patients over 60 years using the chi-square test, Cox regression analysis, and receiver operating characteristics (ROC). The European Society for Clinical Nutrition and Metabolism (ESPEN) guidelines were used as part of the screening of the respective nutritional status of the patients, in particular to filter out malnutrition. Results: A total of 1080 patients were included in this study, whereas 8.05% suffered from malnutrition, defined as a body mass index (BMI) below 18.5 kg/m^2^. The Mini Nutritional Assessment (MNA) screening tool identified the highest proportion of malnourished patients at 14.54%. A total of 36.39% of patients were at risk of malnutrition according to three nutrition scores, with MNA providing the most significant proportion at 41.20%. Patients identified as malnourished had a higher mortality rate, and MNA screening was the only tool to show a significant correlation with postoperative mortality in all survey intervals. The MNA presented the best predictive significance among the screening tools, with a maximum area under the curve (AUC) of 0.7 at 12 month postoperatively. Conclusions: MNA screening has a solid correlation and predictive significance regarding postoperative mortality—therefore routine implementation of this screening in orthopedic/traumatology wards is recommended. Moreover, nutritional substitution therapy can offer a relatively inexpensive and easy-to-implement measure. The Graz malnutrition screening (GMS) shows moderate predictive power and could be considered as an alternative for patients under 60 years of age. A higher albumin level is associated with improved survival probability, but cannot be indicative of nutritional status.

## 1. Introduction

Due to higher life expectancy and an aging population, hip fractures are becoming an increasing problem in our modern society [1]. While hip fractures are rare in young adults and are usually associated with high-impact trauma, such as car accidents, in most older patients, the cause is often a simple fall from a low height [2]. The majority of those affected—about 70% of the patient population—are women with an average age of about 80 years [3].

The costs to the health care system are exceptionally high, and the consequences for those affected are far-reaching [4,5]. Due to the loss of mobility, patients also lose their independence. More than half of the patients do not reach their original level of mobility within the first year after the fracture, and a large proportion remains permanently restricted and dependent on external assistance [6]. The mortality rate after such an injury is also significantly increased. One month after a proximal femur fracture, mortality is approximately 7–10%. Six months after the accident, mortality of up to 20% is already described. After one year, despite intensive management with immediate surgery and rehabilitation, the general mortality increases to 26–36%, depending on the literature [7,8,9]. If another hip fracture occurs within this first year, the probability of survival is notably significantly reduced [10,11].

A common phenomenon among such geriatric patients is poor nutritional status, which is already associated with a more extended hospital stay, more difficult rehabilitation and increased mortality [8,9,12]. Combined with a hip fracture, this leads to an inferior prognosis for those affected [9,13,14].

Since nutritional status is not always clearly evident, especially in older patients, nutritional screening tools offer a cost-effective, quick, and easy remedy [15]. Four of these nutritional scores and two laboratory parameters were investigated in this study for predictive significance concerning postoperative mortality after a surgically treated proximal femur fracture. If high-risk patients can be identified, individualized care can subsequently be provided to these patients to increase the probability of survival.

## 2. Material and Methods

Patients aged over 60 years who experienced a proximal femur fracture and underwent surgical treatment at the Clinical Division of Traumatology, Department of Orthopedics and Trauma Surgery, Medical University of Vienna between January 2018 and November 2019 were included in the investigation. Using retrospective data analysis, the four nutritional screening tools: Mini Nutritional Assessment—Short Form (MNA), Graz Malnutrition Screening (GMS), Nutritional Risk Screening (NRS) and Malnutrition Universal Screening Tool (MUST), as well as individual laboratory parameters (albumin, lymphocytes), were collected as well as matched with postoperative mortality rates at 1, 3, 6, and 12 month/s and examined for an association by chi-square test, Cox regression analysis and calculation of receiver operating characteristics (ROC) and area under the curve (AUC).

Data of routine pre-operative parameters from the day of surgery or one day before surgery were used for the scores. Additionally, the study examined the predictive ability of the scores and their categorization of patients into deceased and alive groups. Mortality data, including the month and year of death, were collected from the Austrian Death Register and linked to mortality in the specified periods. Patients not present in the register were assumed to be alive during data collection.

Surgical treatment was performed according to current guidelines. Intracapsular fractures (medial and lateral femoral neck fractures) were treated using cannulated screws, dynamic hip screws, hemiarthroplasty or total arthroplasty. Extracapsular fractures (per/subtrochanteric fractures) were fixed by intramedullary nailing or, in rare cases, by dynamic hip screws.

Patients treated conservatively or who suffered from periprosthetic or pathological fractures, polytrauma, and patients with insufficient data, impossible to complete the scores were excluded.

After consideration of the exclusion criteria, 1080 patients were included in the study; among them, 69.5% (*n* = 751) were female, with an overall average age of 81.1 years and an average body mass index of 23.8 kg/m^2^. According to WHO criteria, a BMI below 18.5 kg/m^2^ was considered malnutrition [16]. Of the patient population, a total of 8.05% (*n* = 87) suffered from inadequate nutritional status.

The study adhered to ethical guidelines and received approval from the ethics board of the Medical University of Vienna (EK 1655/2020).

### 2.1. Statistical Analysis

Categorical variables were presented numerically, accompanied by their corresponding percentages. In contrast, continuous variables were described using the mean and standard deviation or the median and interquartile range (IQR) for non-parametric data. The normal distribution of the data was assessed using the Shapiro–Wilk test. As applicable, statistical comparisons were performed using appropriate tests, including Student’s *t*-test or chi-square test.

To evaluate the predictive ability of the scores, statistical analyses involved examining the area under the receiver operating characteristic curve for 1-, 3-, 6-, and 12-month mortality predictions. The deviation of the ROC curve from the linear line indicated the assessing system’s predictive ability. A cut-off value of 0.5 was used to categorize patients into predicted deceased and alive groups. Patients with predicted mortality rates exceeding 50% based on any of the scores were grouped as “more likely to die than not”.

Data analysis was conducted using SPSS software for Mac (v.21, IBM Corp., Armonk, NY, USA), and the significance threshold was set at *p* < 0.05.

The chi-square test, Cox regression analysis, and AUC were utilized to assess the association between nutritional screening results and postoperative mortality rates.

### 2.2. Nutrition Scores

#### 2.2.1. NRS (Nutritional Risk Screening)

To be able to use the NRS, one needs the BMI of the patient, information about an unwanted weight loss of more than 5% within a defined period of time, and information about the severity of the current illnesses. Depending on the severity of the condition, 1–3 points are awarded for a mild, moderate, or severe course of the disease. The mild courses include, for example, femoral neck fractures or COPD patients in an acute phase. Illnesses such as stroke, severe pneumonia, or major abdominal surgery are evaluated as moderate disease progression, and severe head injuries or patients requiring intensive care receive 3 points in the survey [17]. Since older age is also associated as a risk factor, all patients ≥70 years receive an additional point in the evaluation.

The points are added up and if the result is ≥3, additional nutritional therapy should be started. The poorer the patient’s nutritional status, the more points are awarded. This shows that both severely malnourished patients without relevant comorbidities and patients in critical illness without relevant malnutrition would benefit from additional nutritional therapy.

#### 2.2.2. MNA (Mini Nutritional Assessment)

The Mini Nutritional Assessment is a nutritional screening specially developed for the elderly population to be used for routine geriatric examinations. It includes specific questions about nutritional and health status, independence, quality of life, cognition, and the subjective feeling of health in the elderly [18].

These questions concern information on reduced food intake (0–2 points) and weight loss (0–3 points) within the last 3 months, information on mobility (0–2 points), and the presence of an acute illness or psychological stress during the last 3 months (0 or 2 points), neuropsychological problems (0–2 points) and BMI (0–3 points). Calf circumference can be used as an alternative to the BMI. The poorer the patient’s nutritional status, the fewer points are awarded. A score of 0–7 unmasks malnutrition, while scores of 8–11 indicate a risk of malnutrition. A normal nutritional status can be assumed at 12–14 points.

#### 2.2.3. GMS (Graz Malnutrition Screening)

The Graz Malnutrition Screening was developed by the multidisciplinary nutrition team of the University Hospital Graz, based on the European guidelines of ESPEN (European Society for Clinical Nutrition and Metabolism) and the already existing screening tools Subjective Global Assessment (SGA) and Nutritional Risk Screening (NRS), to screen patients from different wards in a large hospital to investigate the risk of malnutrition.

In principle, the GMS should be carried out on an interdisciplinary basis. For the first three questions, the nursing staff records the weight loss within the last 3 months (0–2 points), the current BMI (0–2 points), and a reduced food intake, due to loss of appetite/chewing and swallowing difficulties/nausea, vomiting, or diarrhea (1 point each). In the fourth question, the severity of the disease is assessed by a doctor and given 1 or 2 points depending on the degree of severity. Finally, an additional point is added, if the patient is older than 65 years since older age is an independent risk factor for malnutrition.

The points are then added up, whereas a sum of ≥3 indicates a risk of malnutrition [19].

#### 2.2.4. MUST (Malnutrition Universal Screening Tool)

The MUST screening tool was developed to identify (potential) malnutrition in patients within 5 steps. To be able to determine the MUST score, one needs the BMI of the patient, information about unintentional weight loss within the last 3–6 months, and information about the patient’s medical condition in combination with their dietary intake.

The parameters BMI and weight loss can be scored from 0 to 2 points each, and for acutely ill patients, who have not ingested or are expected to ingest orally for more than 5 days, there are 2 additional points. The risk of malnutrition is low with 0 points, with 1 point it is moderate and with 2 points and more the risk for the patient is high. In the case of medium risk, the food intake should be observed and precisely documented for 3 days and based on this an evaluation should be made. Immediate action is required in patients with a score of ≥2 [20].

The screening tool was originally developed to be able to predict not only the nutritional status, but also the outcome of hospitalized adults.

## 3. Results

Among the nutritional scores, the MNA provided the highest proportion of malnourished patients at 14.54% (*n* = 157) followed by the MUST score at 13.52% (*n* = 146).

**Figure 1:**
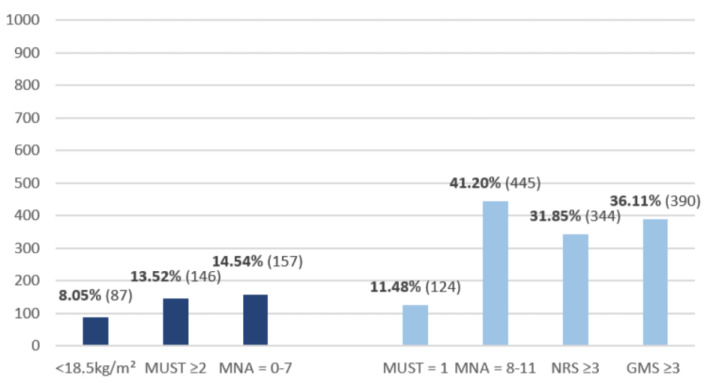
Nutrition Score Assessment: number of patients with malnutrition (left) and a high risk of malnutrition (right).

The risk of malnutrition was identified by the three nutrition scores: MNA, GMS and NRS in an average of 36.39% of patients. The MUST score was not included in this average value as an outlier, with only 11.48%. The MNA again provided the most significant proportion of patients at risk, with 41.20% (Figure 1). 

**Figure 2:**
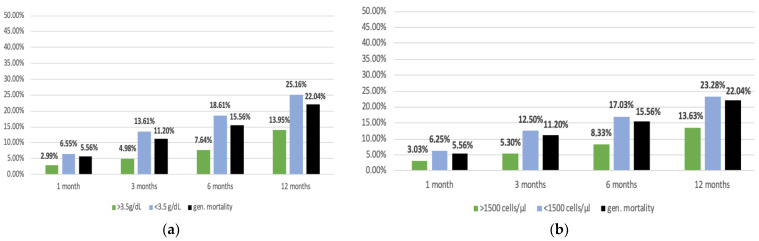
(**a**): mortality in relation to albumin concentration. (**b**): mortality in relation to lymphocyte concentration.

The average serum albumin concentration of the entire study population was 3.2 g/dL. Typically, a serum albumin concentration of <3.5 g/dL indicates malnutrition (22) (Figure 2a). Among the 1080 subjects in this study, 72.13% (*n* = 779) had such values. Since almost ¾ of the entire study population had an albumin level below 3.5 g/dL, the mortality rate was not significantly higher than the general mortality rate. Looking at the postoperative mortality rate as a function of the albumin concentration, it becomes apparent that an albumin concentration >3.5 g/dL acts as a protective factor to a certain extent. The mortality of this patient group was relatively very low and comparable to the survival probability of patients with an MNA score of 12–14 points. A remarkable postoperative survival probability of the patient group above 3.5 g/dL albumin concentration within the first 12 months is striking (*p* < 0.01).

Total lymphocyte concentration showed similar results with a cut-off value of 1500 cells/µL, with 75.76% (*n* = 816) of patients below 1500 cells/μL. A cell count of >1500 cells/µL also seems to act as a protective factor, as mortality in this patient group was relatively low. However, patients with fewer than 1500 lymphocytes/µL did not have a particularly high mortality—it is similar to the overall mortality of the patient collective (Figure 2b).

Overall, MNA screening showed the most impressive results in this work. Patients identified as malnourished had an approximately 5-fold-higher mortality rate compared to those with normal nutrition. From the third postoperative month onwards, the odds ratio was >7 (Table 1).

These results are also reflected in the multivariate Cox regression analysis. MNA screening was the only tool to show a significant correlation with postoperative mortality in all survey intervals (*p* < 0.01). The same applies to the albumin concentration and the body mass index (*p* < 0.01). In the univariate Cox regression analysis, all nutrition scores and both laboratory parameters showed a significant association with higher postoperative mortality in all survey parameters at all times (*p* < 0.05).

**Figure 3:**
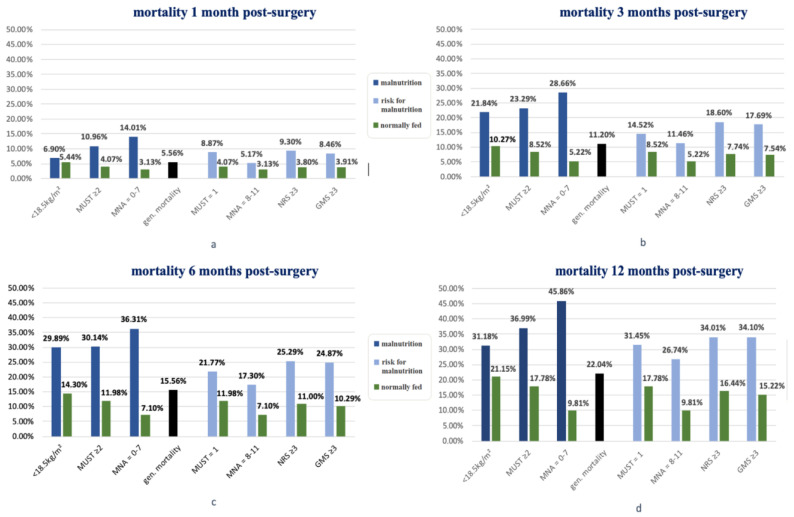
General mortality and nutritional status according to BMI and nutrition scores during survey intervals (**a**–**d**).

When examining the four graphs (Figure 3a–d), the first thing that stands out is the MNA showing the highest mortality rate of malnourished people and the best survival probability of those with normal nutrition in all four time intervals.

The remaining nutritional scores presented relatively similar results. Depending on the survey interval, they alternated with more or less impressive results (Table 1). Compared to all-cause mortality, among the nutritional scores, each group of at-risk patients showed a higher mortality, and each subgroup of those with a normal diet showed a lower mortality rate (Figure 3a–d).

**Figure 4:**
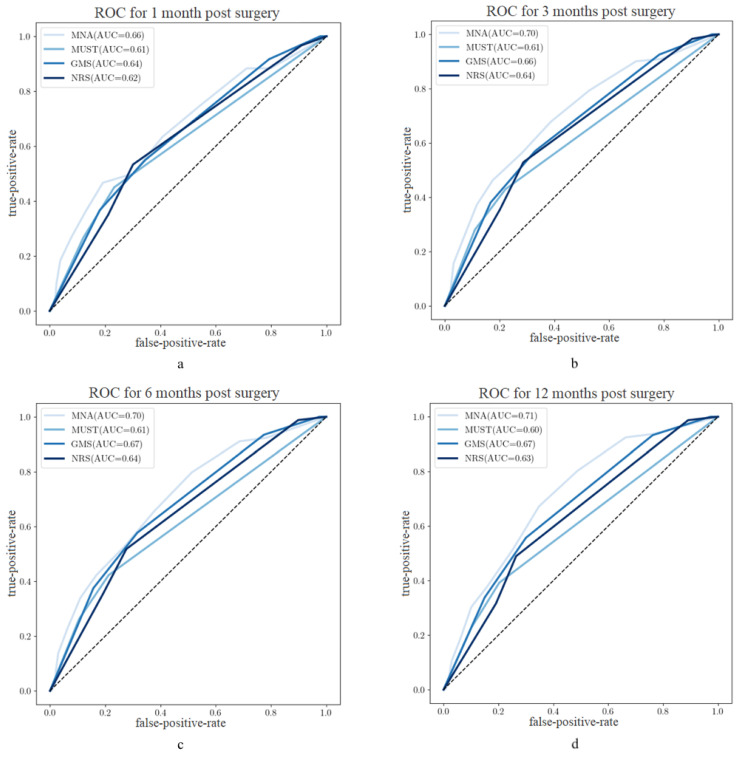
(**a**–**d**): AUC for mortality-prediction in different postoperative periods.

In terms of predictive significance, the MNA presented the best predictive significance among the screening tools with a maximum AUC of 0.71 at 12 month postoperatively, followed by the GMS with a maximum value of 0.67 (Figure 4a–d).

## 4. Discussion

The European Society for Clinical Nutrition and Metabolism (ESPEN) has recommended these screening tools to evaluate patients for possible malnutrition [21]. In principle, they include various combined questions on body mass index (BMI), weight loss, food intake, disease severity and age [22,23]. Among the screening tools mentioned, the MNA was explicitly developed for the elderly population and additionally considered neuropsychological problems and patient mobility in the questions [24,25].

The MNA screening was convincing with its strong correlation regarding postoperative mortality of malnourished patients in this work. In a comparable study by J. van Wissen et al. (23), 226 patients were also assessed by MNA and compared with the mortality register after one year. Except for a higher mortality rate of the normally nourished patients (17% vs. 9.81% in this paper), the mortality of the high-risk and malnourished patients is precisely the same as the results of this paper. Another study [26] with 472 patients examined the same question and obtained a mortality of 37% for malnourished patients four months postoperatively. Patients in the risk group with 8–11 points had a mortality of 26%, and the control group of the normally nourished only had a mortality of 10%. A meta-analysis of four studies [25] also found significantly higher mortality associated with both patients with an MNA of 0–7 points and an MNA of 8–11 points (*p* < 0.001).

A high sensitivity speaks in favor of the use of the MNA. However, screening performs somewhat worse concerning specificity, as the proportion of high-risk patients identified can be too high [18]. In a meta-analysis by Malafarina et al. [9] of a total of 12 studies involving 2195 hip fracture patients, on average, 45.2% were normally nourished (44.26% in this paper), 35.3% were in the at-risk group (41.20% in this paper), and 18.7% were identified as malnourished (14.54% in this paper). Therefore, the retrospective data evaluation of the MNA provides plausible results, even though individual studies with a smaller number of cases may vary more in their breakdown.

However, why does the MNA screening tool stand out particularly firmly in this study with its results among the four nutrition screening tools? The MNA was developed as a screening tool specifically for the older population. The average age of the patients included in this study was 81.1 years. In addition to questions about body weight and unintentional weight loss, the cognitive state and mobility of the patients were also taken into account [17,27]. Existing cognitive impairment associated with a hip fracture is an independent risk factor for increased mortality [28]. In a study of 173 patients [29], hip fracture patients with mild-to-moderate cognitive impairment showed a 5-fold increase in mortality within the first 12 months. In addition, cognitive impairment is often associated with malnutrition, as patients no longer eat enough on their own [30]. Furthermore, since poor functional status prior to hip fracture is also associated with increased mortality, the predictive value of the MNA screening tool further benefits from this [31].

Similar to this work, a study by Koren-Hakim et al. [32] evaluated 215 patients after hip fracture using the MNA, NRS and MUST and examined their postoperative mortality. Only the MNA showed a significant correlation (*p* < 0.001), and the NRS showed a slightly weaker correlation (*p* < 0.05) between higher mortality after 36 months.

An advantage of the NRS is that it produced reliable results, regardless of, whether it was performed by a nurse, a dietician, or a doctor [33].

The MUST screening did not show a clear correlation in this respect.

In the case of the GMS, it has established itself as a validated nutritional screening tool. To check its validity, it was compared with the already validated nutritional scores MNA and NRS and, thanks to very similar measurement results, proved to be a reliable tool for checking adult patients in acute care for the risk of malnutrition. No corresponding literature currently examines the nutrition score for the GMS screening for a possible correlation with postoperative mortality after hip fracture.

In several studies, a low albumin level below 3.5 g/dL has already been associated with higher postoperative mortality and a greater complication rate related to hip fractures [23,25,34,35,36]. As almost 75% of the patient population in this work had a serum albumin concentration of less than 3.5 g/dL, the main focus is on the improved survival probability in patients with higher albumin concentrations of more than 3.5 g/dL. While the mortality of patients with low albumin levels did not differ significantly from the general mortality, higher albumin levels above 3.5 g/dL acted as a specific protective factor (Figure 2a). Since the threshold value of 3.5 g/dL albumin is also used as an indicator for malnutrition [23], no conclusion can be drawn about the nutritional status. However, it can be assumed that patients identified as being at nutritional risk using nutritional screening tools also had a reduced serum albumin concentration. This assumption is supported by a large meta-analysis [23] of 111 studies involving 52.911 subjects examining the association between specific blood biomarkers and malnutrition. Patients identified as malnourished by MNA showed significantly reduced albumin levels (3.3 g/dL) compared to those not at risk (3.8 g/dL). Similar results were obtained by NRS (NRS < 3 points: 3.4 g/dL/NRS ≥3 points: 3.7 g/dL). However, there are also indications in this study that the threshold value of 3.5 g/dL albumin is particularly unsuitable for older people [23]. According to the results, this threshold would lead to an underdiagnosis of malnutrition, as only the most severe cases are identified. The results of this work cannot confirm this assumption. However, it should be noted that the current state of health is a significant factor in the diagnosis of malnutrition using albumin levels. Acute illness, for example, due to inflammation or tissue damage in a hip fracture, has a strong negative influence on serum albumin concentration and can be used as an explanation for the low average concentration [34,35,36].

However, the routine performance of the MNA on orthopedic/traumatology wards is recommended to identify patients at risk and to be able to initiate appropriate therapeutic measures. Nutritional substitution therapy, in particular, offers a relatively inexpensive and easy-to-implement measure. The current state of studies records only weak evidence for the positive effects of such therapy. Future studies could investigate the effects of nutritional substitution, specifically in patients at risk of MNA.

## 5. Conclusions

Considering the results, the MNA screening stands out among the nutritional scores with a solid correlation and an excellent predictive significance regarding postoperative mortality. We recommend routine screening using MNA to identify malnourished and at-risk patients for targeted therapy. This has the potential to help patients in a targeted manner in times of scarce resources.

GMS screening shows the second-best predictive power and could therefore be considered an alternative for use in patients under 60 years of age. In addition, an improved survival probability of patients with albumin values above 3.5 g/dL can be observed. However, no statement about the nutritional status can be derived from this.

## Figures and Tables

**Table 1:** Odds ratio for mortality depending on nutrition score and group; *p*-values are based on chi-square test.

	Malnutrition				Risk of Malnutrition	
	MNA 0–7	MUST ≥ 2	GMS ≥ 3	NRS ≥ 3	MNA 8–11	MUST = 1
1. Month						
postoperative	5.03	3.06	2.34	2.65	1.68	2.29
	Odds Ratio	Odds Ratio	Odds Ratio	Odds Ratio	Odds Ratio	Odds Ratio
	(*p* < 0.01)	(*p* < 0.01)	(*p* < 0.01)	(*p* < 0.01)	(*p* > 0.05)	(*p* < 0.01)
2. Month						
postoperative	7.28	3.48	2.70	2.79	2.35	1.82
	Odds Ratio	Odds Ratio	Odds Ratio	Odds Ratio	Odds Ratio	Odds Ratio
	(*p* < 0.01)	(*p* < 0.01)	(*p* < 0.01)	(*p* < 0.01)	(*p* < 0.01)	(*p* < 0.01)
6. Month						
postoperative	7.44	3.40	2.96	2.74	2.73	2.05
	Odds Ratio	Odds Ratio	Odds Ratio	Odds Ratio	Odds Ratio	Odds Ratio
	(*p* < 0.01)	(*p* < 0.01)	(*p* < 0.01)	(*p* < 0.01)	(*p* < 0.01)	(*p* < 0.01)
12. Month						
postoperative	7.77	2.94	2.93	2.67	3.35	2.12
	Odds Ratio	Odds Ratio	Odds Ratio	Odds Ratio	Odds Ratio	Odds Ratio
	(*p* < 0.01)	(*p* < 0.01)	(*p* < 0.01)	(*p* < 0.01)	(*p* < 0.01)	(*p* < 0.01)

## Data Availability

Data is unavailable due to ethical restrictions.
